# Cyclodextrin carboxylate improves the stability and activity of nisin in a wider range of application conditions

**DOI:** 10.1038/s41538-023-00181-7

**Published:** 2023-05-20

**Authors:** Yao Hu, Kequan Xing, Xiaojing Li, Shangyuan Sang, David Julian McClements, Long Chen, Jie Long, Aiquan Jiao, Xueming Xu, Jinpeng Wang, Zhengyu Jin, Chao Qiu

**Affiliations:** 1 State Key Laboratory of Food Science and Technology, School of Food Science and Technology, Collaborative Innovation Center of Food Safety and Quality Control in Jiangsu Province, Jiangnan University, Wuxi, Jiangsu 214122 China; 2 College of Light Industry and Food Engineering, Nanjing Forestry University, Nanjing, Jiangsu 210037 China; 3 College of Food and Pharmaceutical Sciences, Ningbo University, 169 Qixing South Road, Ningbo, Zhejiang 315832 China; 4 Department of Food Science, University of Massachusetts, Amherst, MA 01060 USA; 5 Beijing Advanced Innovation Center for Food Nutrition and Human Health, China-Canada Joint Lab of Food Nutrition and Health (Beijing), School of Food and Health, Beijing Technology and Business University (BTBU), 11 Fucheng Road, Beijing, 100048 China

**Keywords:** Nutrition, Agriculture

## Abstract

Nisin is a natural bacteriocin that exhibits good antibacterial activity against Gram-positive bacteria. It has good solubility, stability, and activity under acidic conditions, but it becomes less soluble, stable, and active when the solution pH exceeds 6.0, which severely restricted the industrial application range of nisin as antibacterial agent. In this study, we investigated the potential of complexing nisin with a cyclodextrin carboxylate, succinic acid-β-cyclodextrin (SACD), to overcome the disadvantages. Strong hydrogen bonding was shown between the nisin and SACD, promoting the formation of nisin-SACD complexes. These complexes exhibited good solubility under neutral and alkaline conditions, and good stability after being held at high pH values during processing with high-steam sterilization. Moreover, the nisin-SACD complexes displayed significantly improved antibacterial activity against model Gram-positive bacteria (*S. aureus*). This study shows that complexation can improve the efficacy of nisin under neutral and alkaline situations, which may greatly broaden its application range in food, medical, and other industries.

## Introduction

Nisin is a small peptide composed of 34 amino acid residues that is produced by *Lactococcus lactis* subspecies strain, which is the only bacteriocin approved as a food preservative^1^. It is generally recognized as safe (GRAS), and is widely used in the food, medicine, and agricultural industries. Nisin shows broad-spectrum antibacterial activity against Gram-positive bacteria. It is believed to adsorb to the cell membranes of bacteria, disrupt them, and cause internal cellular substances to be released, thereby promoting cell death^2,3^. Under acidic conditions (pH < 6.0), nisin displays desirable solubility and stability with only a slight loss of activity after heat treatment^4,5^. However, the structure of nisin changes under alkaline conditions due to an intermolecular nucleophilic addition reaction, which results in decreased water-solubility, thermal stability, and antibacterial activity^6,7^. Thus, the industrial application of nisin as a natural antimicrobial is currently limited to acidic conditions.

The pH of most physiological fluids is in the range of 6.0–8.5, including intracellular, extracellular, and intestinal fluids^8^. To extend the industrial applications of nisin, efforts have therefore been devoted to identifying strategies to maintain its solubility, stability, and antibacterial activity under physiological situations. Organic acids can associate with nisin in aqueous solutions through hydrogen bonding, which may increase the performance of nisin. For instance, Adhikari et al. ^7^ showed that a nisin-organic acid composite had a much higher antimicrobial activity at pH 8.0 than pure nisin. Using a combination of nisin with EDTA has also been shown to increase the antibacterial activity of nisin^9^, which was attributed to the ability of the chelating agent to increase the permeability of the bacterial cell walls. However, there was no obvious improvement in the stability of the nisin. Other efforts intending to improve the stability of nisin usually rely on nano-delivery systems prepared from biopolymers, such as chitosan, cellulose, and pectin^3,10,11^. However, constructing these delivery systems is often complicated, costly, and difficult to scale-up, which limits their industrial application. Consequently, it would be advantageous to develop a simple and cheap method that could meet practical industrial requirements.

Cyclodextrins (CDs) are cyclic oligosaccharides composed of various numbers of α-D-glucopyranose units, which are produced from starch by enzymatic hydrolysis, and are authorized for utilization in food and health products in most regions of the world^12^. The cyclic nature of CDs leads to the creation of molecules that have a hydrophobic core and a hydrophilic exterior, which makes them suitable as host molecules to incorporate non-polar guest molecules or moieties^13^. Encapsulation of bioactive compounds in CDs often improves their water dispersibility, enhances their resistance to heat, light, and oxygen, and allows for controlled release^14,15^. Previously, researchers have shown that encapsulation of nisin within β-CDs improved its antibacterial activity during the preservation of cooked pork meat^16^, which was attributed to the formation of nisin-CDs complexes that changed the microenvironment of nisin. However, there is still a need for an alternative form of CD that can enhance the solubility, stability, and antimicrobial activity of nisin at the same time.

β-CD displays low cost and strong binding affinity to hydrophobic guest substances among the commonly used cyclodextrins in food industry. However, its water solubility (around 1.85%) is insufficient for many commercial applications^17^. The dose of β-CD used to enhance the solubility of nisin was therefore restricted in practical systems. β-CD derivatives widely used in commercial pharmaceutical applications, including hydroxypropyl-β-CD (HP-β-CD), sulfobutylether-β-CD (SBE-β-CD), and methyl-β-CD (M-β-CD) show greatly improved water-dispersity after chemical modification^17,18^. New strategies for creating food-grade cyclodextrin derivatives are also being explored. For instance, octenyl and octadecenyl succinic anhydrides have been attached to the hydroxyl groups on cyclodextrin molecules to produce derivatives with good emulsifying properties^19,20^. However, such large substituents to β-CD may impact its ability to incorporate guest molecules due to steric hindrance effects. In our previous study, a cyclodextrin derivative, succinic acid-β-cyclodextrin (SACD) was obtained and shown to be over 50-fold more water-dispersible than β-CD. Meanwhile, SACD has a significantly higher complexation behavior to guest molecules^21^. The modification followed a simple and safe chemical dry-heating procedure with all the chemicals used were food grade. The obtained SACD has been demonstrated noncytotoxic, which might be a promising strategy to improve the solubility and bioactivity of nisin.

In this study, we intended to improve the solubility and antibacterial activity of nisin by forming complexes with SACD. The molecular interactions between nisin and SACD were elucidated by analyzing the molecular and crystalline structures of the complexes. The water-solubility and stability of the nisin-SACD complex were measured. Furthermore, the antibacterial activity of nisin-SACD complexes against a model Gram-positive food-borne pathogen (*S. aureus*.) was elucidated.

## Results and discussions

### Structural analysis of nisin-SACD complexes

**Fig. 1:**
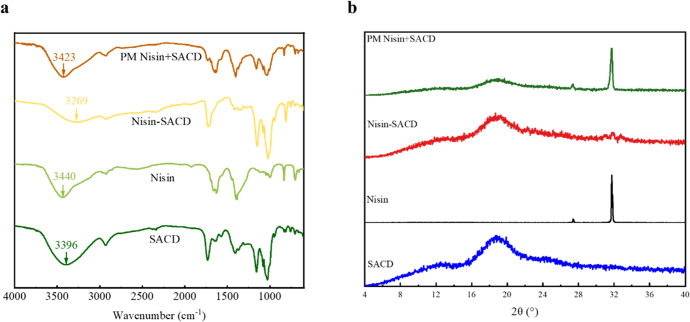
Molecular and crystal structural analysis of nisin-SACD complexes. FTIR (**a**) and XRD (**b**) spectra of SACD, nisin, nisin-SACD, and physical mixture of nisin and SACD (PM nisin+SACD).

In order to reveal the interactions between nisin and SACD driving the formation of nisin-SACD complexes, FTIR analysis was used to compare the chemical properties of complexed nisin-SACD with nisin, SACD, and the physical mixture of nisin and SACD (PM nisin+SACD) (Fig. 1a). The molecular interactions between nisin and SACD were illustrated from the FTIR spectra. The broad peak at around 3369 cm^−1^ was attributed to the O–H stretching vibration of the SACD molecules. The peak at 1732 cm^−^^1^ was attributed to the C=O stretching of the ester bonds of SACD. The characteristic bands at 2929, 1155, and 1027 cm^−^^1^ were attributed to the CH_2_, C–O–C, and C–OH groups of SACD, respectively. The broad peak absorption band of nisin at 3440 cm^−^^1^ was attributed to axial O–H/N–H stretching vibrations, the band at 2923 cm^−^^1^ was attributed to the stretching vibration of C–H, and the band at around 1630 cm^−^^1^ was attributed to the absorption by the amide group^22^. After complexing with SACD, the band corresponding to O–H and N–H significantly shifted to a lower wavenumber (3269 cm^−^^1^), with a wavenumber shift of −127 and −171 cm^−^^1^ in comparison to SACD and nisin, respectively. Meanwhile, a 10 cm^−^^1^ redshift was observed at the ester C=O band for the nisin-SACD at 1722 cm^−^^1^. The above phenomena indicate that strong hydrogen bonding took place between nisin and SACD. Presumably, succinic acid branches on the SACD molecules played an important role in these interactions. Other researchers have also reported strong hydrogen bonding between similar components in complexes^23,24^. The amide band was not observed in the spectrum of the nisin-SACD complexes, which may be a result of the shielding effects of SACD due to the relatively low proportion of nisin in the complex. The FTIR spectrum of the physical mixture of nisin + SACD was consistent with a combination of the two individual components, with no obvious band shifts being observed. These results suggest that complexation did not occur in the simple physical mixtures of nisin and SACD.

It is known that the bioavailability of bioactive substances is directly correlated with the dispersity of them in matrices. In the pharmaceutical industry, physically converting the bioactive substances from crystallized state to amorphous state is regarded as an effective method to improve the dispersity of them in matrices^25^. In previous studies, CDs were frequently complexed with many hydrophobic bioactive compounds including nutraceuticals and pharmaceuticals. After the complexation, the physical state of bioactive compounds converted from crystallized structure to amorphous form with much increased solubility, contributing to effectively enhanced bioavailability and bioactivity of the compounds^26–28^. Therefore, the crystalline structure of nisin was analyzed by using X-ray diffractometer after complexing with SACD in this study, to predict the activity of nisin-SACD complexes. Nisin exhibited diffraction peaks at 2θ values of 27.4° and 31.7° (Fig. 1b), which is consistent with previous studies^29^. No distinct diffraction peaks were observed for SACD, suggesting SACD has an amorphous structure^21^. Only a slight change could be observed after complexing nisin with SACD, suggesting that nisin existed in an amorphous form in the nisin-SACD complexes. Therefore, we speculated that the amorphous nature of the nisin-SACD complexes would increase the biological activity of the nisin. As a comparison, the physical mixture of the nisin + SACD showed a simple combination of diffraction patterns of nisin and SACD, including the sharp crystalline peaks of nisin. These results indicate that the nisin remained in a crystalline form in the physical mixtures, which further demonstrates the successful formation of nisin-SACD complexes.

### Thermogravimetric analysis of nisin-SACD complexes

**Fig. 2:**
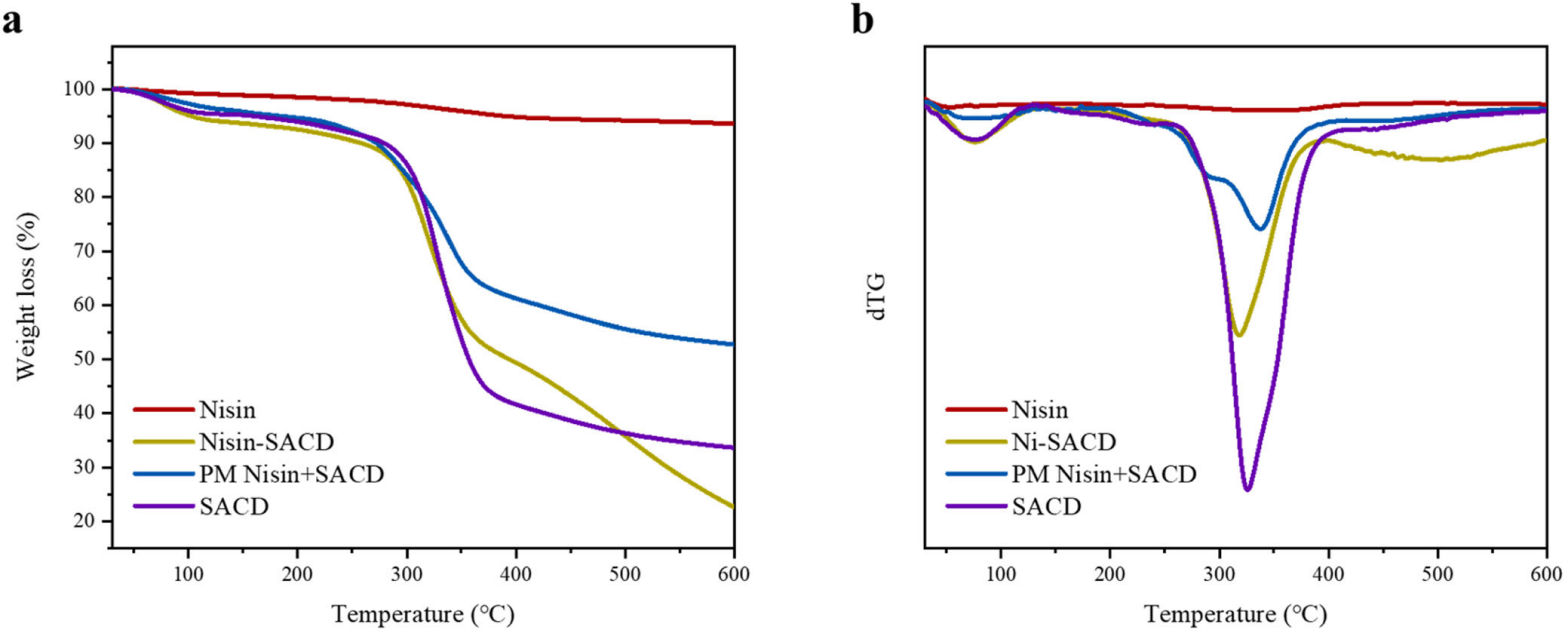
Thermalgravimetric analysis. The TG (**a**) and dTG (**b**) curves of nisin, nisin-SACD, PM nisin + SACD, and SACD.

The complexation of nisin and SACD causes changes in the molecular arrangement and interactions of the nisin, which could affect its thermal stability. Thermogravimetric analysis was therefore carried out to evaluate the mass change behavior of nisin-SACD complexes and mixtures during controlled heating. Generally, the first-stage weight loss observed below 100 °C was attributed to the evaporation of moisture in the samples. As shown in Fig. 2a, there was no obvious weight loss of nisin in the early stage below 100 °C, which suggests that little water was trapped in the powdered nisin due to its poor hydrophilicity at neutral pH. Moreover, there was only a slight weight loss (<7%) observed for nisin by the end of the heating process (600 °C), which could be due to its high degree of crystallinity. Other researchers have reported similar stability of nisin under dry-heating conditions^30^.

For SACD, two distinct stages of mass loss during heating were observed in the TG curves. The first stage was attributed to moisture evaporation due to the presence of water molecules trapped in the hydrophilic structure of SACD, which contributed to about 4.5% of the total weight loss. At the end of heating, nearly 67% of weight loss had occurred for this sample, which was mainly attributed to thermal degradation of the SACD molecules at around 300 °C and higher.

For nisin-SACD complexes, the moisture loss during the first stage of heating was slightly less than for pure SACD, which might be because a certain amount of the hydroxyl and carboxyl groups on the SACD were occupied by nisin and therefore not available for water molecules to adsorb to. There was also a steep decrease in mass around 300 °C, which was mainly attributed to the thermal degradation of the SACD. At higher temperatures, the rate of thermal degradation was faster for the nisin-SACD complexes than for pure SACD, which may have been because the molecular forces were weaker.

Compared to nisin-SACD complexes, less weight loss of the physical mixture of nisin and SACD was displayed at the end of the heating process. It might be because the free nisin part that with highly stable crystalline structure. It suggests that the dry-heating stability of samples reflected and proved specific intermolecular reactions in nisin-SACD complexes. However, the results did not correlate with the biological activity of nisin directly, for peptide structure change could happen even with an insignificant mass loss^31^.

To better understand the dry-heating process, the mass change rate curves of the samples were calculated from the TG curves (Fig. 2b). The temperatures where the fastest thermal degradation took place could easily be observed in the resulting dTG profiles. The maximum decomposition peak of nisin occurred at 333 °C, which suggests that a small amount of weakly crystallized nisin degraded at this temperature. The maximum decomposition peak of SACD occurred at 326 °C, with most of this substance being degraded at this temperature. For the nisin-SACD complex, a lower decomposition temperature of 317 °C was observed, which is consistent with weaker molecular forces being present within their amorphous structures. Interestingly, a broad peak was observed at around 504 °C for the nisin-SACD complexes, which might be due to the progressive thermal degradation of the SACD in the complexes. The physical mixtures of nisin and SACD exhibited a maximum decomposition rate at around 337 °C, which was attributed to the thermal degradation of the SACD.

### Solubility of nisin-SACD complexes

**Fig. 3:**
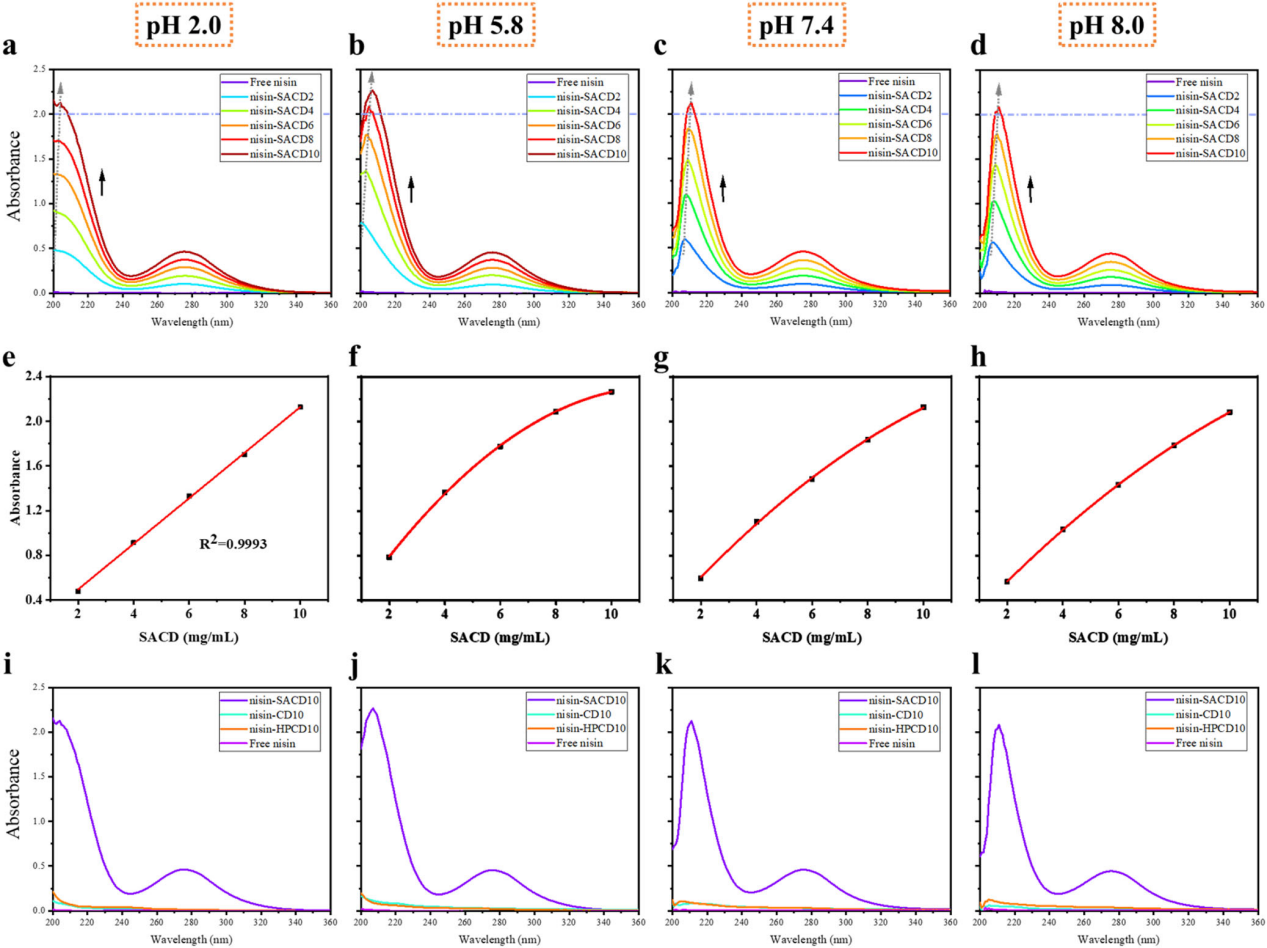
The complexation behavior of nisin and SACD in solutions. The UV-visible spectra of nisin-SACD with various concentrations of SACD (2, 4, 6, 8, and 10 mg/mL) at pH 2.0 (**a**), pH 5.8 (**b**), pH 7.4 (**c**), and pH 8.0 (**d**); the solubility fitting curve of nisin-SACD at pH 2.0 (**e**), pH 5.8 (**f**), pH 7.4 (**g**), and pH 8.0 (**h**); and the UV-visible spectra of free nisin and complexed nisin with SACD, HPCD, and CD and at pH 2.0 (**i**), pH 5.8 (**j**), pH 7.4 (**k**), and pH 8.0 (**l**).

The solubility and complexation behavior of nisin and SACD were investigated through UV-vis spectra analysis (Fig. 3). The maximum absorbance of pure nisin occurred at around 201 nm, which is related to its peptide backbone secondary structure^32^. However, this peak could not be observed in the UV spectra because the nisin concentration was too low. For the SACD, a peak was observed at 280 nm, which can be related to the absorption of unsaturated branches on the SACD molecules^33^. After being complexed with SACD, the absorbance of nisin in aqueous solutions increased significantly with increasing SACD concentration. It suggests the gradually enhanced solubility of nisin. In general, nisin was recognized as soluble under acidic conditions. It was observed that the solubility of nisin even increased significantly by SACD at pH 2.0 (Fig. 3a), suggesting the occurrence of microenvironmental change of nisin. The maximum absorbance wavelength was shifted from 201 to 204 nm gradually with increasing SACD concentrations. Such a redshift phenomenon demonstrates increased hydrophobic interaction force between nisin and SACD, which contributes to enhanced environmental non-polarity of nisin^16^. When the pH increased to 5.8, the absorbance of nisin in solutions exhibited higher values at the same SACD concentration (Fig. 3b). The maximum absorbance of nisin gradually redshifted to 207 nm as the SACD concentration was increased. These results suggest that the complexation of nisin and SACD became stronger as the pH increased. More intense interactions between the two components could be observed at pH 7.4 and 8.0 that were known as less advantageous conditions for nisin to solubilize^5^. Even higher redshift phenomenon to 211 nm was displayed at both alkaline situations (Fig. 3c, d). Though the maximum absorbance of nisin slightly dropped down, the solubility of nisin still kept at high levels in comparison to pure nisin in the two alkaline situations.

The solubility curves of nisin in SACD solutions were plotted at different pH values (Fig. 3e–h). At pH 2.0, the absorbance of the nisin increased linearly with rising SACD concentration. The relative hydrophilic property of nisin under pH 2.0 might be a benefit for forming complexes with SACD, for more solubilized nisin existed in the complexing solutions. While at higher pH of 5.8, 7.4, and 8.0, non-linear fitted curves were observed for the solubility of nisin in SACD solutions. This effect might be attributed to intermolecular nucleophilic addition reactions happened to nisin, which reduced the solubility of nisin at alkaline pH^34^. Fortunately, SACD displayed satisfying effects hindering the undesired transformation of nisin under relative alkaline conditions, endowing nisin to be soluble in the applied pH range.

To distinguish the effectiveness of SACD in broadening the application range of nisin, we further compared the complexation behavior of nisin with SACD and two commonly used CDs, β-CD and HP-β-CD (Fig. 3i–l). Both β-CD and HP-β-CD show considerable improvements on the solubility of nisin. Obvious redshifts for the wavelength at the maximum absorbance were observed at relative alkaline conditions (e.g., the maximum absorbance of nisin in HP-β-CD solution appeared at 205 nm at pH 8.0). The absorbance of nisin in solutions containing HP-β-CD were higher than that containing β-CD for the four pH situations determined. However, the maximum absorbance values of nisin in SACD solutions were far beyond the value of nisin in either β-CD or HP-β-CD solutions. Therefore, it could be concluded that SACD displayed some advantages in promoting the solubility of nisin at neutral and alkaline situations. These results indicate that complexing nisin with SACD can greatly increase its water dispersibility, which would be beneficial for wider utilization of nisin in various industries.

### pH stability of nisin-SACD complexes

**Fig. 4:**
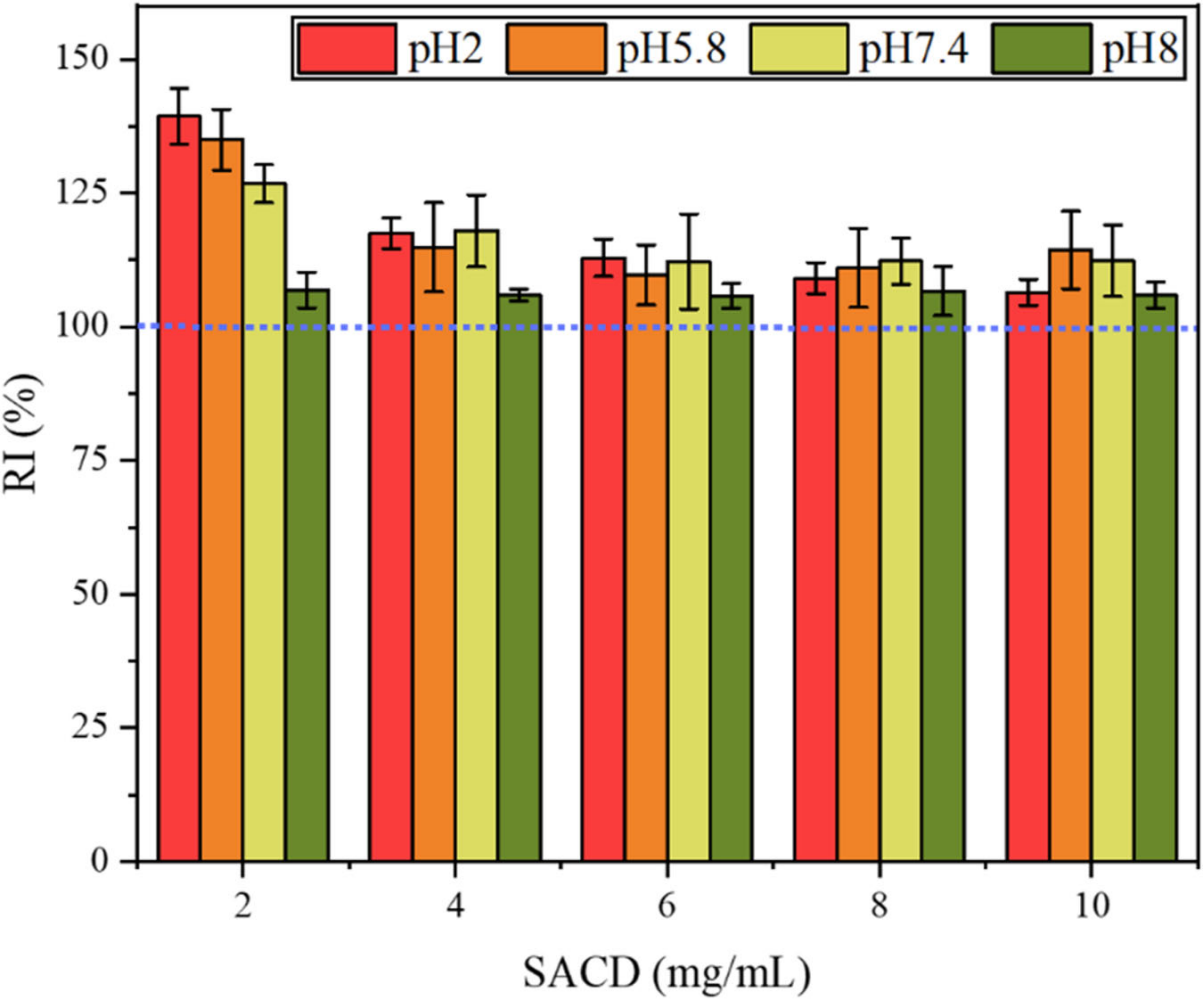
pH does not affect the stability of nisin-SACD complexes in solution. The retention index (RI) of nisin-SACD complexes at different SACD concentrations (2, 4, 6, 8, and 10 mg/mL) after keeping at pH 2.0, 5.8, 7.4, and 8.0 for 10 days. The error bar represents the standard deviation from three tests of the same sample.

The stability of nisin-SACD complexes at different pH values was evaluated by measuring the solubilized nisin in solutions after 0- and 10-day storage. The retention index (RI) was calculated from this data and used to indicate the stability of the nisin in the different complexes (Fig. 4). All the samples displayed good stability after storing for 10 days, with RI values above 100%. This result suggests that complexation was able to stabilize the nisin at all pH values studied, and that the formation of complexes may have been relatively slow. Notably, RI values of the nisin in the complexes containing low SACD concentrations were appreciably higher than those containing high concentrations. According to a previous study, there are multiple ester branches in one SACD molecule, which can bind to nisin as shown by FTIR analysis^21^. As a peptide with a relatively disordered molecular structure, it is hard for nisin to completely complex with relatively small SACD molecules. It takes time for nisin to extend and find those unoccupied sites on the surfaces of the SACD molecules. When more SACD was included in the systems, less increase was observed for the RI of nisin. It may be because there were fewer free sites on the nisin molecule available to bind to the SACD.

### Sterilization stability of nisin-SACD complexes

**Fig. 5:**
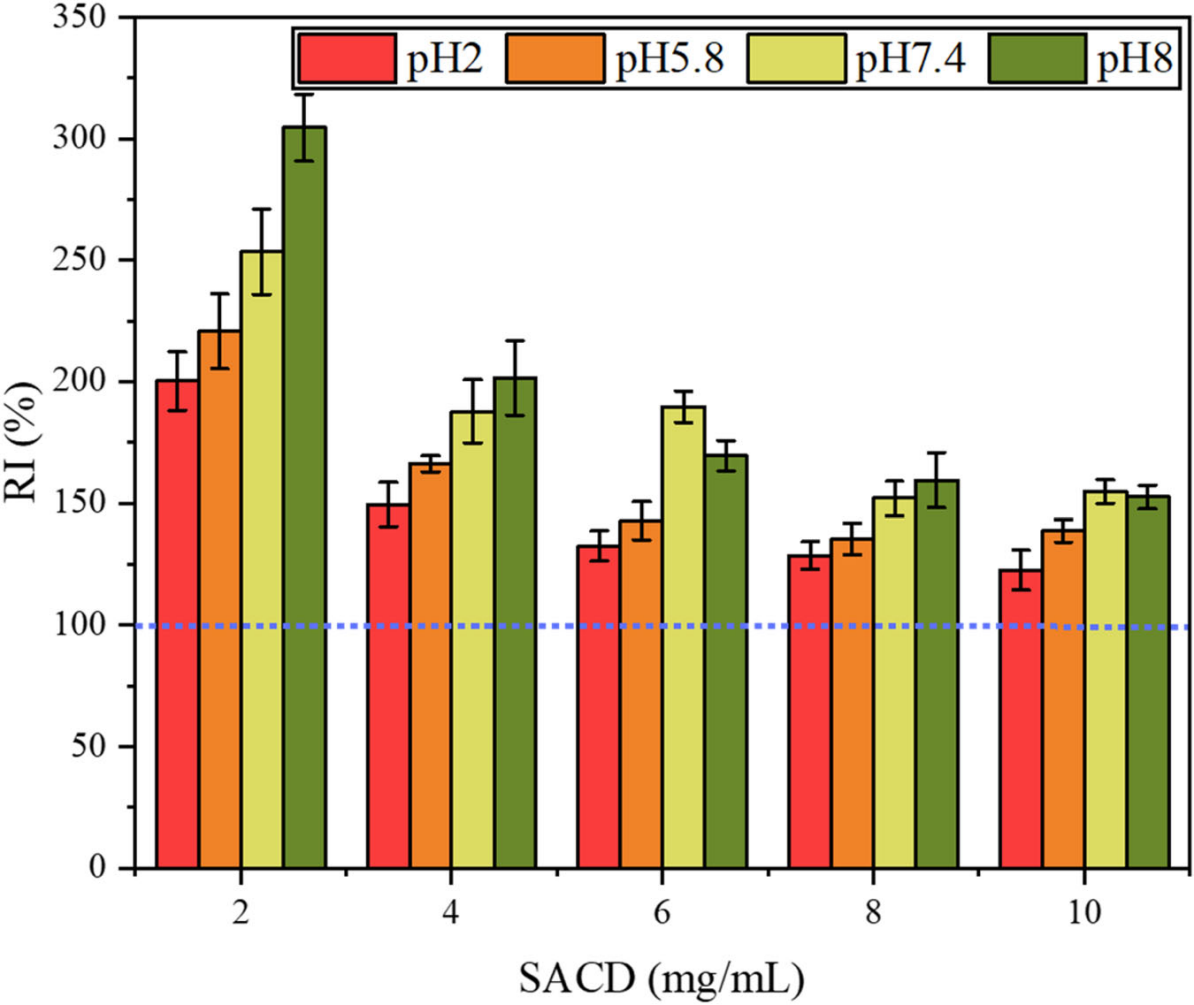
Sterilization does not affect the stability of nisin-SACD complexes in solution. The RI of nisin-SACD complexes with different SACD concentrations (2, 4, 6, 8, and 10 mg/mL) and pH (2.0, 5.8, 7.4, and 8.0) situations after treating at 121 °C for 30 min. The error bar represents the standard deviation from three tests of the same sample.

The resistance of the nisin-SACD complexes to high-temperature steam sterilization was determined because the thermal stability of formulations is important for industrial applications. Previous studies have shown that chemical degradation of nisin occurs when it is thermally processed^34^, which reduces its antibacterial activity^3,35^. After complexing with SACD, no thermal degradation of nisin was observed at any pH studied and there was an appreciable increase in the amount of solubilized nisin after the thermal treatment (Fig. 5). These effects were most pronounced for the complexes containing low concentrations of SACD (2 mg/mL), which displayed more than two times of solubilized nisin compared to those without sterilization. The solubilization effect became more noticeable at higher pH values, with up to three times more nisin being solubilized in the 2 mg/mL SACD solution after the thermal treatment. When more SACD molecules were present during the complexation process, the enhancement of high temperature on the solubilization of nisin seemed reduced, which could be because the saturation of the complexes between nisin and SACD only occurred gradually. Even so, an appreciable increase in the RI value for nisin (>120%) was still observed at a SACD concentration of 10 mg/mL. In a previous study, the complexation of nisin with gum Arabic and pectin protected it from thermal degradation at 121 °C, but the solubility of nisin was not improved^1,3^. These results suggest that complexation with SACD not only increased the thermal stability of nisin, but also increased its solubilization during heating.

### Antibacterial effect of nisin-SACD complexes

**Fig. 6:**
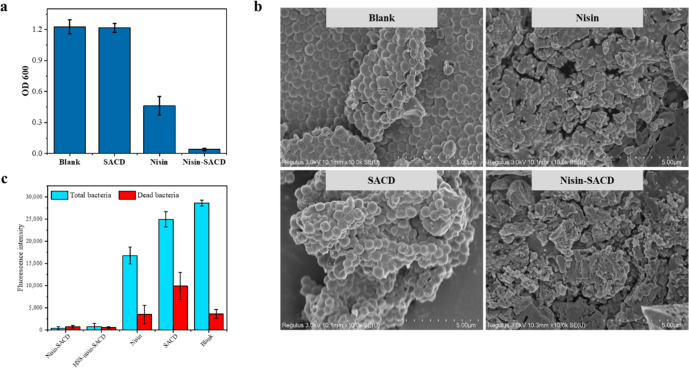
Antibacterial effect of nisin-SACD complexes to *S. aureus*. The OD_600_ values of the bacterial cultures without any treatment and treated with SACD, nisin, and nisin-SACD complexes (**a**). The SEM images of *S. aureus* collected from cultures of the blank, SACD, nisin, and nisin-SACD complexes samples (**b**). The fluorescence intensity of live/dead bacteria in *S. aureus* cultures treated with blank, SACD, nisin, and nisin-SACD complexes samples (**c**). The error bar represents the standard deviation from three tests of the same sample.

Finally, we determined the antibacterial activity of the nisin-SACD complexes developed in this study against *S. aureus*. *S. aureus* is commonly used as model Gram-positive bacteria to predict the antibacterial effects of the bacteriostatic agent. Meanwhile, it is a significant cause of foodborne illness leading to gastroenteritis, diarrhea, and vomiting^36^. Foods susceptible to *S. aureus* intoxication including meat, egg, and dairy products^37^. It is known that nisin can deactivate bacteria by adsorbing to the cell membrane, disrupting the cell membrane, and promoting the loss of essential intracellular components^38^. As shown in Fig. 6a, pure nisin exhibited only moderate antibacterial activity under the conditions used, with the OD_600_ value of the bacterial suspension decreasing from 1.23 to 0.46 (2.7-fold). To observe the cell membrane change of bacteria after treating with nisin-SACD complexes, the surface microstructure of treated bacteria was observed by collecting SEM images (Fig. 6b). The appearance of the bacteria transformed from sphere to irregular shape, proving the broken of cell membrane. In contrast, much stronger antibacterial activity was exhibited by the nisin-SACD complexes, with the OD_600_ value decreasing to 0.04 (31-fold). Nearly no cell-like structures could be observed in the SEM image for the collection of nisin-SACD complexes treated cultures. This increased antimicrobial activity can be attributed to the increased solubility and chemical stability of the nisin in the complexes. In addition, the increased bactericidal effect of the complexes might also be due to some antibacterial activity of the SACD. SACD contains carboxyl groups at its surfaces that could create a more acidic microenvironment around the bacteria, thereby hindering their growth^27,39^. In our previous study, pure SACD at a relatively high dose did show antibacterial activity against *S. aureus*^27^. Our results, therefore, suggest that the presence of SACD improved the antibacterial effects of nisin by forming complexes that increased its solubility and activity.

To support the practical application of nisin-SACD complexes, the minimum inhibitory concentration (MIC) of nisin-SACD and HSS-nisin-SACD were determined. The two complexes had MIC of 20 mg/mL and 40 mg/mL against *S. aureus*, respectively. However, no significant antibacterial effects were observed at the setting concentration range for using nisin and SACD alone. Though higher MIC was observed for HSS-nisin-SACD, complexation with SACD still shows significant protection and preservation for the biological activity of nisin after high-temperature treatment. After treating the bacteria with nisin-SACD or HSS-nisin-SACD at their MIC levels, both total and dead bacteria existed in low levels in comparison to the untreated group (Fig. 6c). It indicates the proliferations of bacteria were effectively prohibited. Certain degree of inhibition on bacteria proliferation was observed by simply treating the bacteria culture with nisin or SACD as demonstrated earlier. The results again confirmed that the complexation between nisin and SACD exhibited a synergistic effect and significantly enhanced the biological activity of nisin and SACD. Similar synergistic effect existed between nisin and carvacrol was reported by Li et al. ^40^.

In this study, SACD was used to improve the solubility, stability, and antibacterial activity of nisin by forming molecular complexes. Strong interactions were inferred between the nisin and SACD molecules due to the appreciable redshifts of specific peaks observed in the FTIR spectra. The highly crystallized molecular structure of nisin was transformed into an amorphous structure after forming the nisin-SACD complexes. The weaker molecular interactions in the complexes than in pure nisin reduced their overall thermal stability. Nevertheless, the nisin within the complexes appeared to remain stable to degradation at higher temperatures. UV-visible spectroscopy suggested that the interactions between the nisin and SACD depended on the pH of the surrounding solution, with stronger interactions occurring at higher pH values. These results suggest that the complexation of nisin with SACD could improve its solubility, stability, and activity under neutral and alkaline situations. In addition, complexation improved the resistance of the nisin to high steam sterilization. Finally, the antibacterial activity of nisin-SACD and HSS-nisin-SACD complexes was shown to be much effective than pure nisin against a model Gram-positive bacterium (*S. aureus*). Overall, our results indicate that the complexation of nisin with SACD has great potential for improving its utilization as an antibacterial agent in food or pharmaceutical formulations. In future, in vitro and in vivo studies are required to assess the safety and efficacy of these complexes. Moreover, it will be necessary to overcome any legal and scale-up hurdles before they can be used widely for practical applications.

## Methods

### Materials

Nisin from *Streptococcus lactis*, β-cyclodextrin (≥99.5%, β-CD), succinic acid (SA), and sodium hypophosphite (SHP) were purchased from Aladdin Regent Co. (Shanghai, China). Other chemicals were of analytical grade. Deionized water was used to prepare aqueous solutions in all experiments.

### Preparation of SACD

SACD was prepared using the method described in our previous study^21^. Briefly, 4.00 g of β-CD, 2.96 g of SA, 4.00 g of SHP, and 40 mL of deionized water were mixed and stirred until fully solubilized. After pouring the mixed solution onto a circular plate (diameter 160 mm), the sample was dried in an oven at 100 °C for 3–5 h. Then, the plate was transferred into another oven set at 140 °C and kept for 20 min to allow the esterification reaction to occur. After cooling the esterified samples to room temperature (around 0.5 h), the crude product was then collected by firstly solubilizing in 20 mL deionized water and then precipitated by adding an excess volume of absolute ethanol. This washing process was repeated another two or three times to ensure no impurities remained in the final product. After which, the sample was dried overnight to remove the ethanol and the final product was collected.

### Preparation of nisin-SACD complexes

Nisin was complexed with SACD by solubilizing 1 mg of nisin within 10 mL of SACD solution (400 mg/mL). After thoroughly mixing, the solution was centrifuged at 10,000 rpm for 5 min to remove insoluble substances. The supernatant was collected and freeze-dried to convert it into a powdered form for solid-state assessment.

### Solubility test

The impact of pH on the solubility of nisin in SACD solutions was evaluated. Solutions with SACD concentrations of 0, 2, 4, 6, 8, and 10 mg/mL were prepared by solubilizing SACD in buffer solution with different pH values. The resulting samples are referred to as free nisin, nisin-SACD2, nisin-SACD4, nisin-SACD6, nisin-SACD8, and nisin-SACD10, respectively. Except when using HCl to adjust the pH to 2.0, phosphate buffer solutions were used to prepare different pH values (5.8, 7.4, and 8.0). Then, 1 mg of nisin was added to the above SACD solutions with different pH values. The SACD concentration ranged from 2 to 10 mg/mL at each pH value. The resulting suspensions were stirred for 8 h at 25 °C for the nisin to reach saturated solubilization. After removing any undissolved substances by passing through 0.45 μm pore size filters, the solubilized amount of nisin was quantified using an UV-visible spectrophotometer (UV–1800PC, Mapada, China) at 201 nm. The host-guest interactions between nisin and SACD could be observed from the wavelength shifts of maximum absorbance of nisin after being complexed with SACD. The solubility curves of nisin in SACD solutions at different pH values were then constructed by plotting the absorbance value as a function of SACD concentration. Additional control groups using nisin complexed with 10 mg/mL of β-CD and 10 mg/mL of HP-β-CD were prepared at pH 7.4 to prove the effectiveness of nisin-SACD. The samples are referred to as nisin-CD10 and nisin-HPCD10, respectively. All the other procedures used for preparing the complex solutions were the same as for the nisin-SACD samples.

### FTIR analysis of nisin-SACD

Infrared spectra of the powdered samples were collected using a FTIR spectrometer (Nicolet Nexus 470, Thermo Electron Corporation, Waltham, MA, USA) at wavenumbers from 400 to 4000 cm^−1^.

### X-ray diffraction analysis

The crystalline structure of the powered nisin-SACD complexes was determined using X-ray diffraction analysis (XRD) utilizing a commercial instrument (D2 PHASER, Bruker, Germany) at an operating voltage of 40 kV and a diffraction angle (2θ) range from 4° to 40°.

### Thermogravimetric analysis of nisin-SACD

The thermal behavior of the nisin-SACD complexes was evaluated using a thermogravimetric (TG) analysis system (TGA2, Mettler–Toledo, Schwerzenbach, Switzerland) under N_2_ (50 mL/min) at a 10 °C/min heating rate over a temperature range from 30 to 600 °C.

### pH and heat stability of nisin-SACD

To evaluate the effects of pH on the stability of nisin-SACD, the amount of nisin in the complex solutions was determined 10 days after solubilization. The storage duration was set based on preliminary experiments ensuring the complexation process reached full equilibrium. The thermal stability of the nisin-SACD complexes was also assessed by exposing them to a high-pressure steam sterilization treatment at 121 °C for 30 min. The fraction of nisin solubilized within the nisin-SACD complexes was determined by measuring the absorbance at 201 nm using an UV-visible spectrophotometer (UV–1800PC, Mapada, China). As a control group, the same procedures were carried out on a suspension of nisin. The retention index (RI) was then calculated using the following equation:

1 $${{{\mathrm{RI}}}}\left( {{{\mathrm{\% }}}} \right) = \frac{{{{{\mathrm{The}}}}\;{{{\mathrm{absorbance}}}}\;{{{\mathrm{value}}}}\;{{{\mathrm{after}}}}\;{{{\mathrm{the}}}}\;{{{\mathrm{heating}}}}\;{{{\mathrm{process}}}}}}{{{{{\mathrm{The}}}}\;{{{\mathrm{absorbance}}}}\;{{{\mathrm{value}}}}\;{{{\mathrm{before}}}}\;{{{\mathrm{the}}}}\;{{{\mathrm{heating}}}}\;{{{\mathrm{process}}}}}} \times 100{{{\mathrm{\% }}}}$$

### Antimicrobial activity of nisin-SACD complexes

The antimicrobial activities of the samples were determined using *S. aureus* (ATCC 6538) as a representative Gram-positive bacterium. The optical density (OD) was used to assess the antibacterial activity of the nisin and the nisin-SACD complexes^41,42^. The bacteria were precultured on Luria-Bertani media at 37 °C for 24 h to obtain seed cultures. These seed cultures were then diluted to 10^6^ CFU using Luria-Bertani media. 1 mg of Nisin, 50 mg of nisin-SACD (containing 1 mg of nisin), and 50 mg of SACD were weighed and added into 10 mL diluted bacteria culture separately. A bacteria culture containing no sample was used as a blank control group. After incubating at 37 °C for 24 h, the growth of bacteria in the culture media was determined by recording the OD at 600 nm (OD_600_) using an UV-visible spectrophotometer (UV–1800PC, Mapada, China).

After confirming the antibacterial activity of nisin-SACD complexes, the MIC of nisin-SACD complexes was further measured by the broth microdilution method as reported by Li et al. ^40^. The initial concentration of nisin, SACD, nisin-SACD, and high-pressure steam sterilized nisin-SACD (HSS-nisin-SACD) were set as 400 µg/mL, 40 mg/mL, 40 mg/mL (containing 400 µg/mL nisin), and 40 mg/mL (containing 400 µg/mL nisin), respectively. The initial solutions were then serially diluted until the concentration of them reached 3.125 µg/mL, 0.3125 mg/mL, 0.3125 mg/mL, and 0.3125 mg/mL, respectively. 100 µL of the samples at different concentrations were then cultured with 100 µL *S. aureus* dilutions containing 10^6^ CFU bacteria in 96-well plates. The plates were incubated at 37 °C for 24 h. The concentration at which OD_600_ was not significantly different from that of the blank group (without bacterial inoculation) was regarded as the MIC. *S aureus* treated with nisin-SACD and HSS-nisin-SACD at MIC were further stained using a live/dead bacterial double stain kit (LMAI Bio, China), and the number of the total bacteria and dead bacteria could be represented by the fluorescence intensity value directly. Note there is no statistical relationship between the fluorescence intensity of the total bacteria and the dead bacteria, since they are stained by two different fluorescent dyes.

### Morphological analysis of bacteria treated with nisin-SACD complexes

After culturing with nisin-SACD, SACD, and nisin, the morphology of bacteria was visualized using a scanning electron microscope (SU8100, SEM, Hitachi, Japan). The samples were deposited on carbon black tape and then coated with gold before analysis.

### Statistical analysis

All measurements were conducted on separate samples in triplicate. Results that gave numeric values are presented as means ± standard deviations. ANOVA analysis of the experimental data was carried out using SPSS 20 statistical software (SPSS Inc., Chicago, USA). Differences were considered at a significance level of 95% (*p* < 0.05).

### Reporting summary

Further information on research design is available in the Nature Research Reporting Summary linked to this article.

## Supplementary information

Reporting Summary

## Data Availability

The authors declare that all data supporting the findings of this study are available in the paper.
